# Diagnostic biomarkers for α-synucleinopathies- state of the art and future developments: a systematic review

**DOI:** 10.1186/s13024-025-00914-0

**Published:** 2025-12-04

**Authors:** V. Donadio, M. Ingelsson, G. Rizzo, A. Furia, A. Incensi, C. Delprete, M. Pinho, R. Liguori, S. Pritzkow

**Affiliations:** 1 IRCCS Istituto delle Scienze Neurologiche di Bologna, Italia, UOC Clinica Neurologica, Bologna, Italy; 2 Dipartimento di Scienze Biomediche e Neuromotorie, Università di Bologna, Bologna, Italy; 3 Krembil Brain Institute, University Health Network, Toronto, Ontario Canada; 4 Departments of Medicine and Laboratory Medicine & Pathobiology, Tanz Centre for Research in Neurodegenerative Diseases, University of Toronto, Toronto, Ontario Canada; 5 Department of Public Health and Caring Sciences, Molecular Geriatrics, Rudbeck Laboratory, Uppsala University, Uppsala, Sweden; 6 Department of Neurology, University of Texas Mcgovern Medical School at Houston, Houston, TX USA

**Keywords:** Biomarkers, α-synucleinopathies, Skin biopsy, Immunofluorescence, Seed amplification assay

## Abstract

**Background:**

Alpha-synucleinopathies are common disorders that are expected to become increasingly prevalent in the future along with the longer life expectancy. However, their diagnosis is problematic as they are mainly based on clinical criteria without the support of disease-specific biomarkers. This leads to frequent misdiagnoses, as underlined by autopsy studies, and an imprecise selection of patients for clinical trials, preventing progress in the development of disease-modifying treatments. In recent years important advances have been made regarding the development of specific biomarkers for the detection of pathological α-synuclein (α-syn), which may improve the diagnosis of patients affected by α-synucleinopathies.

**Results:**

In this review, we describe in detail the most promising techniques to detect pathological α-syn in patient-derived samples. In particular, we describe the diagnostic accuracy of each individual cerebrospinal fluid (CSF), plasma and skin α-syn biomarker in differentiating α-synucleinopathies from controls, from other neurodegenerative disorders and between different α-synucleinopathies. Furthermore, we underline the main advantages and limitations of these techniques for clinical practice. Finally, we provide our suggestions for further development considering both technical aspects and large-scale standardization.

**Conclusions and Relevance:**

We conclude that immunofluorescence on biopsied skin tissue and the seed amplification assay on CSF show the best diagnostic accuracy and reliability in the studies that have been performed to date. We discuss the opportunities of these techniques as well as the main current limitations and technical problems that need to be considered before they can be adopted for clinical use.

**Graphical Abstract:**

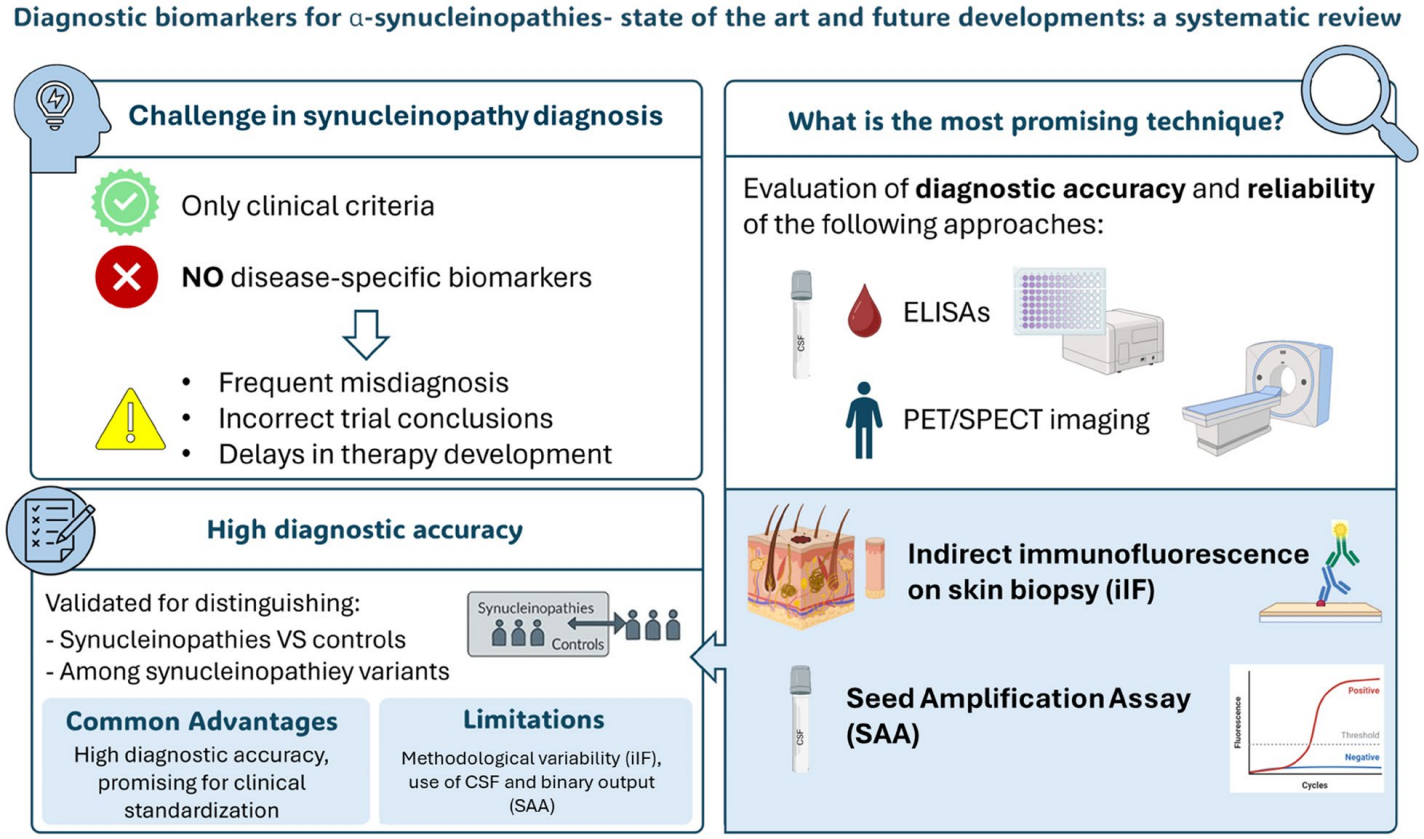

## Background

Alpha-synucleinopathies are neurodegenerative disorders characterized by abnormal deposition of misfolded α-synuclein (α-syn) in neurons or glial cells. [1, 2] In Parkinson’s disease (PD) and dementia with Lewy bodies (DLB), these aggregates appear in cortical and brainstem neurons as Lewy bodies [3] and in peripheral autonomic nerves as Lewy neurites. [4] In pure autonomic failure (PAF) there is an isolated involvement of autonomic nerves in the peripheral nervous system (PNS), which may progress to the central nervous system (CNS). [5] Multiple system atrophy (MSA) is typically characterized by deposition of α-syn as glial cytoplasmic inclusions both in the CNS (in oligodendrocytes [6]) and the PNS (in myelinated Schwann and Remak cells [7]).

Parkinson’s disease is the second most common neurodegenerative disease, with an estimated prevalence of approximately 1% of individuals > 60 years of age [1], whereas DLB is the second most common form of neurodegenerative dementia following Alzheimer’s disease (AD). In addition, the prevalence and incidence of α-synucleinopathies will probably increase along with longer life expectancy and it has been estimated that the number of PD patients will more than double by 2030. [1]

**Fig. 1:**
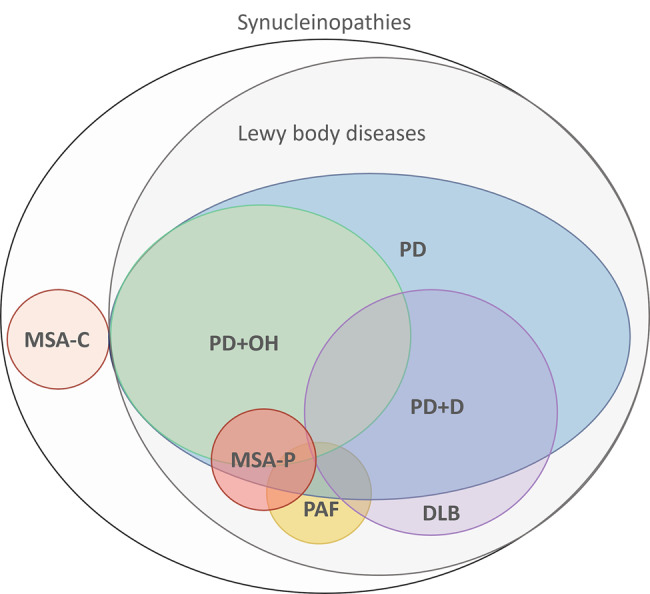
Representation of clinical variants of α-synucleinopathies. Alpha-synucleinopathies are neurodegenerative disorders characterized by fibrillar aggregates of the α-synuclein protein within neurons and glial cells. They include relatively common disorders, such as Parkinson’s disease (PD) and dementia with Lewy bodies (DLB), as well as more rare disorders, such as pure autonomic failure (PAF) and multiple system atrophy (MSA). Especially early in the disease course, these disorders exhibit a large clinical overlap of motor, autonomic and cognitive disturbances, especially early in the disease course, which highlights the need for specific diagnostic biomarkers with a high specificity. PD+D = PD with dementia; MSA-P= multiple system atrophy, parkinsonian variant; MSA-C= multiple system atrophy, cerebellar variant

Despite α-synucleinopathies representing increasingly frequent pathologies, their diagnoses still remain problematic as they are mainly based on clinical criteria. The main symptoms of these disorders include motor and autonomic disturbances, as well as cognitive decline, which show varying degrees of overlap among the different clinical forms, making it difficult to identify a single clinical entity based solely on symptomatology (Fig. 1). The absence of reliable biomarkers for these pathologies leads to frequent misdiagnosis, underlined by autopsy studies reporting that the clinical diagnosis of PD is incorrect in about 25% of patients [8]. Moreover, 20% of patients with a clinical diagnosis of MSA were found instead to have PD or DLB at autopsy. [9] In addition, DLB can often be misdiagnosed as AD or other forms of dementia. Furthermore, up to 15% of patients entering clinical trials for early untreated idiopathic PD may not exhibit dopaminergic defects in the basal ganglia neither at baseline nor at follow-up, which may suggest alternative diagnoses. [10] Misdiagnoses may result in delayed treatment or increased risk of drug-related adverse events and can also lead to a wrongful inclusion of patients into clinical trials, preventing progress in the development of disease-modifying treatments.

Current evidence points to α-syn deposition being the primary pathogenic factor of neurodegeneration in α-synucleinopathies. [11] Therefore, detecting abnormal α-syn aggregation, particularly at the early disease phases when the neurodegeneration is still limited, represents the most promising approach to identifying disease-specific biomarkers for these disorders. This is especially important because α-synucleinopathies may present prodromal signs such as rapid eye movement sleep behavior disorder (RBD) and olfactory impairment, which may precede a full-blown clinical picture by several years. However, these symptoms are not specific of an underlying α-synucleinopathy as RBD can be secondary also to other conditions (e.g., narcolepsy, antidepressant therapy, diencephalic lesions) [12] and olfactory loss can occur in different neurodegenerative diseases (e.g. tauopathies such as AD, TDP-43 proteinopathy as amyotrophic lateral sclerosis or Huntington’s disease). [13]

Thus, validated and robust pathological biomarkers could improve diagnostic certainty in differentiating α-synucleinopathies from healthy controls and other neurodegenerative disorders.

## Detection of in vivo α-synuclein

In the last years important advances have been made in the development of biomarkers able to detect pathological α-syn in the living patient. In the following paragraphs we review different approaches and discuss their strengths and limitations.

### Search strategy and inclusion criteria

Published studies were identified from the following sources: (a) National Library of Medicine’s MEDLINE database; (b) Elsevier’s EMBASE database; (c) Web of Science. The analysis was conducted until April 27^th^ 2025.

The search strategies include the use of the following syntax:

- ELISAs, mass spectrometry, PET, SPECT, IMC, iIF, PMCA, RT-QuIC, Seed Amplification Assay, Skin biopsy, Skin samples, CSF, blood AND
- α-syn, native α-synuclein, synuclein seeding activity, oligomer α-synuclein, phosphorylated α-synuclein, α-synucleinopathy, PD, MSA, RBD, DLB, PAF, RBD.

Criteria for study selection and consideration were: in vivo detection of misfolded α-synuclein; original studies; case-controls design; accepted clinical diagnostic criteria for α-synucleinopathies. Outcome measures considered included: diagnostic performance parameters, reliability parameters, diagnostic accuracy parameters. Position papers and case reports were not considered.

## ELISAs, mass spectrometry and conformational antibodies in biological fluids

### Cerebrospinal fluid

Large efforts have been made to measure total levels of α-syn in patient biofluids with enzyme linked immunosorbent assays (ELISAs), but results have been inconsistent. [14] A reduced level in PD compared to control cerebrospinal fluid (CSF) has been the most common finding, although several investigations have failed to find group differences. [14] Twenty-one out of 35 published cohorts with at least ten patients and ten controls showed decreased CSF total α-syn (sensitivities 56–94%, specificities 25–98%) in PD vs controls, with increased levels in one cohort and no difference between PD and control in the remaining cohorts. [14]

Also, detection of abnormal α-syn species has been explored. Studies based on ELISAs using the same antibody for capturing and reporting to detect oligomeric α-syn showed higher CSF oligomer levels in PD compared to controls. [15] Six out of seven cohorts have displayed slightly increased CSF oligomeric α-syn (sensitivities 65–89%, specificities 83–91%). [14] When measuring α-syn phosphorylated at serine 129 (*p*-α-syn), higher CSF levels have been found in PD compared to MSA and control (sensitivity 86%, specificity 30%). [16]

Several meta-analyses have been performed. [17, 18] For the 35 total α-syn cohorts, Cohen’s d (a measure used to indicate the standardized difference between two means) was −0.46 (SE = 0.08; *p* < 0.001). For the seven cohorts studying oligomeric α-syn, Cohen’s d was 1.24 (SE = 0.46; *p* = 0.01). [14]

Also for other α-synucleinopathies results have been conflicting, although several studies have indicated lower CSF α-syn in DLB and MSA compared to controls [19] as well as in PD and PDD compared to both controls and DLB [20], although no differences were found between PD and MSA. [21]

Taken together, regardless of the examined CSF α-syn species, there are substantial overlaps in the levels between patients and controls. However, most studies have indicated that lower CSF total α-syn can be detected in PD with a fairly high sensitivity but a rather low specificity. Thus, normal levels can rule out an α-synucleinopathy with a certain precision, whereas decreased levels can not discriminate between patients and controls or between patients with the various such diseases. Targeting post-translationally altered protein forms may offer better opportunities. Additional studies on larger cohorts are warranted to evaluate whether certain forms of oligomeric α-syn or *p*-α-syn could become useful CSF-based diagnostic markers for PD and other α-synucleinopathies.

### Blood

Most studies have reported increased plasma levels of total α-syn in PD and MSA patients. [18] However, decreased levels in α-synucleinopathy patients compared to healthy controls have also been reported. [22] Similar to CSF, α-syn oligomers were found to be increased in PD compared to healthy control plasma [23], although other studies did not find any difference between patients and controls. [24, 25] In addition, no differences in either total or oligomeric α-syn could be found, whereas plasma levels of *p*-α-syn were found to be slightly higher in PD than in controls. [26, 27] However, another study did not find any group differences. [28]

Also for blood-based α-syn, a meta-analysis has been performed. When including 32 studies on 2683 PD patients and 1838 controls, overall total α-syn levels in PD plasma or serum were increased (SMD = 0.85, *p* = 0.004). However, no differences could be observed for oligomeric (five studies, SMD = 1.40, *p* = 0.347) or phosphorylated (four studies, SMD = 0.57, *p* = 0.925) α-syn between PD and controls. [24] An additional problem is that even those studies that found significant differences at the group level (patients vs controls) showed a substantial overlapping between individual patients and control, limiting their use for patient diagnosis.

Other approaches have focused on particular blood cells, but neither analyses of leucocytes, lymphomonocytes, mononuclear cells nor platelets have shown any alterations in α-syn levels between patients and controls. [29] In addition, CNS-derived extracellular vesicles have been explored. Since the first published paper, which indicated robustly increased levels of α-syn in L1CAM-positive PD plasma exosomes [30], most studies have failed to demonstrate any clear differences compared to controls. When including eleven such studies in a recent meta-analysis a significant increase in PD could be found (Std.MD = 2.48, 95% CI = 0.57–4.41, *p* = 0.01), although it should be noted that most studies showed no or only minimal group differences. [31] Also when meta-analyzing five studies on oligomeric α-syn, higher levels were found in PD than in controls (Std.MD = 3.36, 95% CI = 1.69–5.58, *p* = 0.0003), although three of the studies did not show any such differences. [31]

Taken together, it has not yet been possible to find any robust blood-based α-syn diagnostic biomarkers. The lack of reliable results may be influenced by technical obstacles, such as potential blood contamination with α-syn from erythrocytes, which could account for the conflicting data. Additional efforts to measure pathogenically modified α-syn species in either plasma, serum or in exosomes of specific CNS origins may represent successful future strategies to develop a blood-based biomarker for the α-synucleinopathies.

## PET/SPECT imaging

The availability of positron emission tomography (PET) tracers that can detect α-syn aggregates in vivo represents an unmet clinical need. It is challenging due to different structures of α-syn aggregates, the frequent coexistence with other pathological protein deposits such as amyloid-β (Aβ), and their relatively low amounts in the brain. Thus, high-affinity ligands are needed and some are currently in the pipeline, e.g., [11C]MODAG-001 [32], [18F]F0502B [33], and [18F]SPAL-T-06. [34] Interestingly, the latter displayed a distinct uptake in the putamen and pons as well as in the cerebellar white matter and peduncles of two MSA patients. Another ligand, [18F]ACI-12589 [35], demonstrated a clear binding in the cerebellar white matter and middle cerebellar peduncles of MSA patients, but showed limited binding in PD. For yet another ligand, [18F]C05-05, a recent study reported an increased signal in the midbrain of PD and DLB patients, as well as in the putamen of a MSA-P patient. [36] The failure of the trials conducted so far is most likely due to the fact that the highest concentration of α-syn is intracellular and therefore difficult to detect by tracers administered intravenously.

Future scientific research efforts should focus on the identification of ligands that can easily cross the blood-brain barrier and bind with high affinity and selectivity to the intracellular disease-related pathological α-syn aggregates in both PD and MSA patients.

## Indirect immunofluorescence in skin samples

**Fig. 2:**
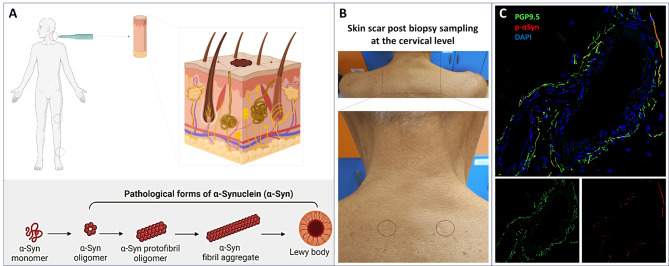
Illustration of the skin biopsy to perform the iIF technique. **A**) analyses of abnormal accumulations of phosphorylated α-synuclein (α-syn) should be performed in skin samples from both proximal (such as the cervical region C7) and distal (such as the thigh and/or leg) sites. **B**) the skin biopsy can be taken under local anaesthesia by a 3 mm diameter punch, resulting in only minor discomfort to the patient. The biopsy does not require sutures and only leaves a small, barely visible scar (arrows). **C**) confocal microscopy (x400) images of phosphorylated α-syn deposits in skin nerves of a patient with pd. Abnormal α-syn aggregates were found in autonomic fibers of the deep derma. Autonomic nerve fibers identified by PGP 9.5 were found around an arteriole (**C**). Most of the PGP 9.5 immunoreactive fibers displayed phosphorylated α-syn (C^I^) as neuritic inclusions demonstrated by the merged image (C^II^). Created with BioRender.com

The possibility of identifying skin *p*-α-syn by immunohistochemistry (IHC) using a bright-field microscopy was first reported in 2010. This is a simple and fast technique, but the positivity rate obtained in patients with α-synucleinopathy has been low, with data limited to autopsy cases. [37] In addition, IHC presents important limitations as it does not allow for the combination of signals, thereby preventing co-localization studies and the fixation process tends to over-fix the tissue, probably reducing peripheral nerve immunostaining. [38, 39] These limitations have been overcome by indirect immunofluorescence (iIF). This technique to detect *p*-α-syn in skin biopsies from living patients was first described in 2013 by the Bologna skin lab (Fig. 2). [4] Since the first report, numerous articles from different centers have shown that the technique has a high diagnostic accuracy in differentiating patients with α-synucleinopathy from healthy controls. [40–43]

The skin biopsy is generally performed using a 3 mm punch biopsy under sterile conditions after local anaesthesia and usually without any sutures. The skin samples are usually fixed in cold 4% paraformaldehyde or Zamboni’s fixative (4% paraformaldehyde plus picric acid) for a period ranging from 30 minutes at room temperature [43] to 4 °C overnight (approximately 18 hours). [4, 40–42] Freezing is not required and samples can therefore be stored at 4 °C. In the original description of this technique two skin samples for each skin site were analyzed to increase the likelihood of detecting *p*-α-syn [4], but in the majority of the following studies only one single skin sample was analyzed for each skin site. [41–44] The skin sections are cut frozen at different thicknesses (10 or 50 μm). The thickness may influence the *p*-α-syn positivity, as 50 μm sections have shown higher positivity than those at 10 μm because a larger skin volume leads to a higher probability of detecting the patchy *p*-α-syn deposition. [44]

Different commercially available primary antibodies raised against *p*-α-syn have been used. The best yield has been seen with monoclonal antibodies from either rabbit or mouse [39, 43], whereas polyclonal antibodies frequently lead to non-specific signals. As reported in the original Bologna skin lab description, *p*-α-syn should be co-stained with a neuronal marker, usually against the pan-neuronal protein gene product PGP 9.5 [4], or NCAM expressed in skin glial non-myelinating Schwann cells, called Remak cells. [7] The co-staining allows for differentiation of pathological misfolded α-syn from a false signal arising from the background staining. As secondary antibodies, those associated with cyanine dye fluorophores or Alexa Fluor(R) are preferably used, allowing for visualization with a confocal microscope mounting appropriate fluorescent filters. The use of distinct fluorophores enables co-localization studies, by combining different primary antibodies without retrieval methods. [4, 7, 40, 41, 43] The primary staining can be improved by using PK [45] or it can be visualized using a more complex retrieval technique such as formic acid or citrate buffer, in conjunction with a biotin-streptavidin amplification. [42, 44] Different methods of analysis to quantify the load of misfolded *p*-α-syn have been described, mainly including the *distribution coefficient* (DC), which calculates the *p*-α-syn location across the various skin sites biopsied) [42], the *fiber rate*, the number of *p*-α-syn fibers in relation to the total number of annexes analyzed or the *absolute number of positive p-α-syn fibers* [46]

The reliability of iIF as a diagnostic tool using patient skin samples has been supported by a high inter- and intra-laboratory reproducibility in disclosing *p*-α-syn positivity. [47, 48] The skin sites typically analyzed with iIF to reveal *p*-α-syn are spine (C7), thigh and leg. [39–43, 46, 47] However, in a recent study it was shown that the cervical and leg punches were sufficient to detect *p*-α-syn with high diagnostic accuracy in MSA and in the Lewy body diseases PAF, PD and DLB. [7]

Available data demonstrate that the analysis of *p*-α-syn in skin biopsies with iIF gives a high diagnostic accuracy in differentiating patients with α-synucleinopathy from healthy controls [40–45] or tauopathies. [49] In particular, a specificity of nearly 100% compared to healthy controls makes this technique an ideal tool for identifying patients with suspected α-synucleinopathies. A high specificity was also obtained when healthy centenarians were analyzed. [50] Importantly, numerous iIF studies demonstrated that skin *p*-α-syn can also be detected at the prodromal stages, e.g. in idiopathic RBD, which can precede the onset of CNS involvement by several years [51, 52].

Considering the high number of papers and the extensive experience, it can be concluded that iIF for the detection of cutaneous *p*-α-syn represents a reliable and reproducible method with high diagnostic accuracy for the diagnosis of α-synucleinopathies and the differentiation of distinct variants of α-synucleinopathies. [7, 42, 46] In addition, this technique presents the advantage of a relatively low cost and an easiness to perform that can be repeated in long-term studies with only minor discomfort and few side-effects for the patient. [53] The main current limitation of iIF of *p*-α-syn is its methodological variability across laboratories and the lack of an automated quantitative method. For robust clinical standardization, differences in skin samples processing (including the thickness of the skin slices, primary antibody and signal development method), as well as the location and number of biopsy samples, should be minimized. In addition, quantification of *p*-α-syn load requires improvement through the implementation of an automated detection method.

## Seed amplification assay

The Seed Amplification Assay (SAA) is a platform technology that detects small amounts of misfolded protein aggregates in biological samples, taking advantage of the unique characteristic of these aggregates to act as seeds to induce misfolding and aggregation of the monomeric form of the protein. [54] The technology was originally developed for replicating infectious prions and was termed PMCA (Protein Misfolding Cyclic Amplification) [55] and later modified and referred to as RT-QuIC (real time-quaking induced conversion). [56] Many investigators have extensively used the PMCA/RT-QuIC method worldwide to detect prions in biological fluids and tissue samples as well as to study the unique biological features of infectious prions. [57] As a result, PMCA and RT-QuIC are now routinely utilized worldwide for diagnosing prion diseases. To avoid confusion with their use for replication of infectious prion material, the terms PMCA and RT-QuIC are now used only for prion detection and the new unified name SAA is used for the assay detecting other non-infectious protein aggregates. [54, 58] The SAA reaction is performed in a cyclical manner with each cycle composed of phases of polymer elongation followed by fragmentation. Typically, patient samples containing minute amounts of misfolded aggregates are incubated as seeds with an excess of monomeric protein as templates (e.g. α-syn) to induce the growth of the polymers. In the second phase, the sample is subjected to mechanical fragmentation to break down the polymers, multiplying the number of seeding-competent nuclei. [54]

**Fig. 3:**
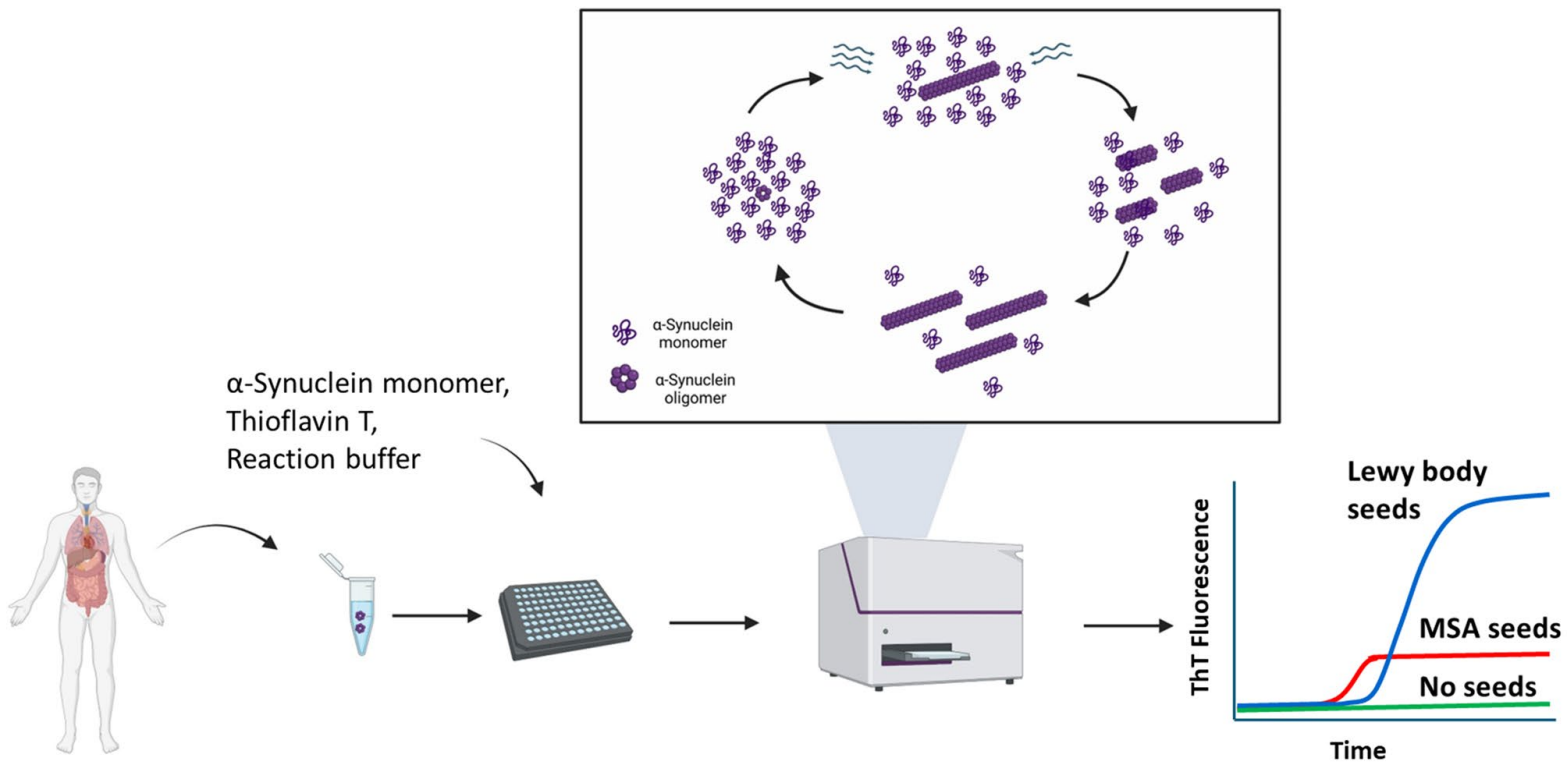
Schematic representation of the SAA reaction. Patient samples containing trace amounts of misfolded aggregates are incubated with a reaction buffer containing thioflavin t, and an excess of α-syn monomeric protein to facilitate polymer growth. The process alternates between phases of polymer elongation and fragmentation, with Mechanical disruption increasing the number of seeding-competent nuclei. Thioflavin T fluorescence is monitored over time to assess the formation of protein aggregates templated by the α-syn seeds present in patients’ samples

The α-syn-SAA is the most advanced of the various SAAs outside the prion field (Fig. 3). The first articles were published online in 2016 [59, 60], and since then many studies by different research groups have reported a high accuracy of α-syn-SAA to detect pathological α-syn aggregates in various biological samples. [54] Most studies have used CSF, because it is a relatively clean liquid that surrounds the brain, which contains the highest amount of α-syn deposits. To date, several thousand CSF samples have been tested from individuals affected by diverse forms of α-synucleinopathies. On average, the assay performs with sensitivities and specificities in the range of 85–95%. [54, 61] Importantly, studies done with the same samples provided blinded to investigators in different laboratories reached very similar results in terms of positive/negative samples despite the fact that distinct laboratories used different SAA protocols. [58, 62] These findings provide strong validation of the assay’s robustness. Strikingly, various studies have reported the detection of α-syn aggregates in CSF of prodromal cases of synucleinopathies, including RBD, PAF and mild cognitive impairment. [63–66] These results indicate that α-syn-SAA has the potential to identify individuals with α-syn pathology much earlier, before the onset of clinical symptoms. Recently, Siderowf and colleagues reported the analysis of CSF samples from 1123 participants, including patients diagnosed with sporadic PD, genetic forms of PD linked to LRKK2 and GDA mutations (including normal carriers), subjects with RBD and controls. [67] The results obtained in this large study indicated that α-syn-SAA can discriminate PD patients from controls with high accuracy. Furthermore, the assay provided information regarding the heterogeneous disease presentation observed in different patient populations. LRRK2 PD patients do not always exhibit accumulation of α-syn in the brain measured histopathologically. Interestingly, SAA also produced a lower rate of positive results in LRRK2 carriers, comparable to the neuropathological analysis. [67] Finally, the study of people affected by RBD showed that the assay can detect prodromal individuals with a high accuracy, long before the onset of clinical disease. In a follow-up of this study, longitudinally collected samples from prodromal PD cases, including patients who did not convert into PD and others that converted at different times after sample collection, were analyzed. The results showed that < 10% of patients with negative α-syn-SAA at baseline converted to PD, whereas > 70% of the SAA-positives converted into clinical disease. [68] Remarkably, analysis of the kinetics of aggregation in the α-syn-SAA reaction showed that the rate of phenoconversion correlates with the speed of seeded aggregation. From the fast seeders, 82.5% of patients converted within 8 years after sample collection, while only 33% of the slow seeders had converted at this time. [68]

Interestingly, recent reports have provided evidence for the ability of α-syn-SAA to discriminate between patients affected by Lewy body diseases (including PD and DLB) and MSA. [69, 70] Using various biochemical, biophysical, structural and biological assays, it was shown that the properties of the aggregates amplified from PD/DLB or MSA CSF samples were clearly different. [69] Comparison of the properties of aggregates seeded with PD/DLB or MSA samples using a variety of techniques showed that the aggregates correspond to different structures. [69] These findings are supported by recent studies by Goedert, Scheres, and colleagues, which show that α-syn extracted from the brains of PD and MSA patients has completely different three-dimensional structures. [70, 71] Our interpretation of the distinction observed in SAA between these samples is that the binding of thioflavin T, used to monitor protein aggregation, to α-syn aggregates from PD and MSA differs due to structural variations, resulting in either lower or higher fluorescence. These findings suggest that α-syn aggregates in different α-synucleinopathies correspond to distinct conformational strains and, importantly, that α-syn-SAA may facilitate the differential diagnosis of distinct diseases caused by α-syn pathology.

Despite the accuracy and robustness of α-syn-SAA, the technique at the present time has some limitations. Up until now, α-syn-SAA has been primarily used as a detection platform producing a binary outcome (positive or negative). This is usually perceived as a limitation of the technology, although the principles underlying SAA suggest that the technique can be made quantitative, and several recent reports have indeed described strategies to obtain quantitative information from α-Syn-SAA analysis. [69, 72, 73] Nevertheless, a complication for employing the kinetic parameters of SAA for quantification encounter the problem that distinct SAA performed in different laboratories with diverse protocols, produced data that cannot be directly comparable. Another important limitation is the use of CSF, which must be collected via lumbar puncture, a relatively invasive procedure. Several articles have reported pilot data supporting the use of α-syn-SAA in samples of skin, olfactory mucosa, saliva, and blood. [74–78] Although, these reports are encouraging, there have not yet been yet many studies to assess the robustness of assays using samples other than CSF, and they have not yet been analytically and clinically validated. Moving forward, the field should establish the appropriate protocols to use SAA in less invasive biological fluids and conduct thorough analytical and clinical validation of these tests.

## Use of the pathological α-synuclein biomarker in clinical practice

Alpha-synucleinopathies represent a group of pathogenetically complex neurodegenerative diseases, as they involve not only the central nervous system but also the peripheral nervous system. Furthermore, the genetic component may play a significant role in the manifestation of the disease, and the presence of co-pathologies is not uncommon. The biological complexity of these disorders was recently highlighted in PD by Höglinger and co-authors [79], who proposed the SynNeurGe system as well as the NSD-ISS staging system proposed by Simuni and colleagues. [80] The proposed systems rely on a biological classification and staging framework for PD, as they define the disease not only through clinical features but also by the in vivo detection of pathological α-syn and the documentation of pathogenic gene variants that either cause or predispose to PD. In line with SynNeurGe and NSD-ISS definitions, we believe it is important to include pathological α-synuclein findings in the diagnostic criteria for PD and α-synucleinopathies, in order to define these diseases on a biological—and not only clinical—level.

The usefulness of considering a specific biomarker for α-synucleinopathies is not limited to the diagnostic definition of these disorders, but should also be extended to other aspects such as measuring disease progression and prognosis, as well as improving the accuracy of clinical trials.

### Clinical assessment

**Fig. 4:**
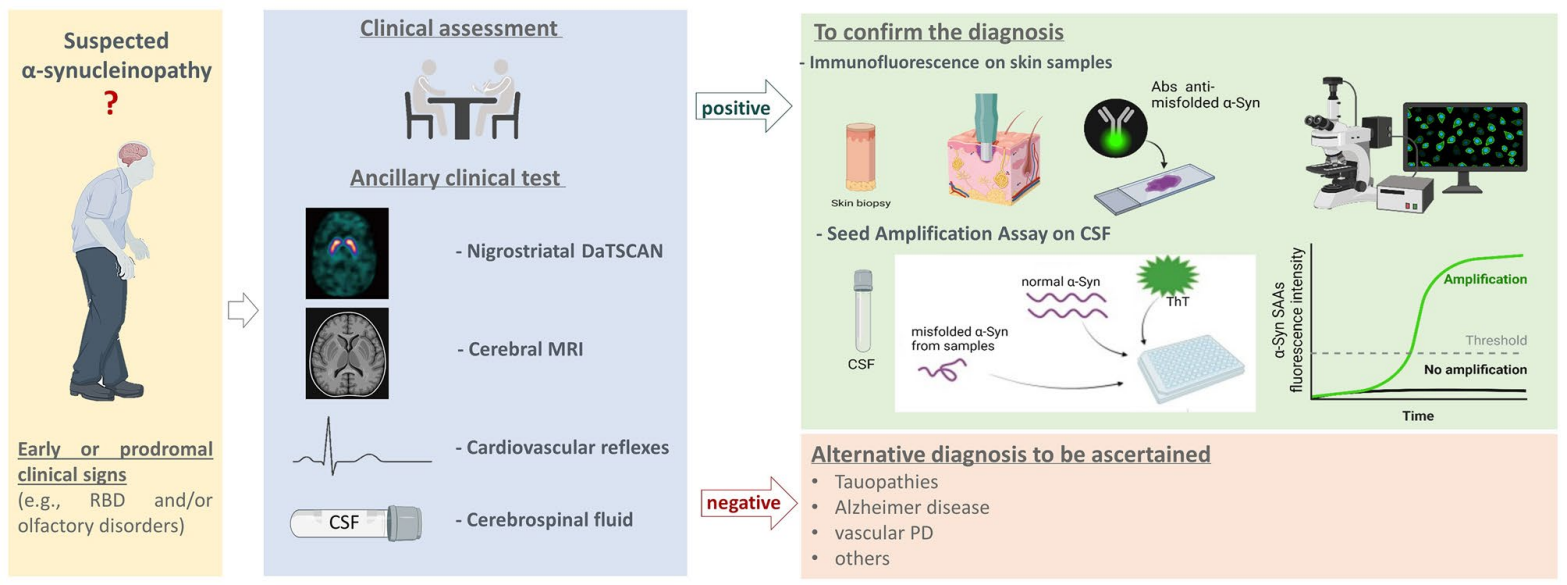
A possible diagnostic flow chart for the clinical use of iIF and SAA. A proposed clinical flow chart for the use of iIF for the detection of phosphorylated α-syn in skin samples and of CSF α-syn-SSA in patients with suspected α-synucleinopathy (i.e. PD, PAF, MSA or DLB), including subjects with prodromal signs and symptoms of such disorders (i.E RBD or hyposmia). Created with BioRender.com

Figure 4 shows a diagnostic flow chart on how skin iIF and α-syn-SAA, that have demonstrated the highest reliability in detecting in vivo pathological α-syn, could be applied in clinical practice. As suggested by Höglinger et al. [79] and Simuni et al., [80] we recommend associating the detection of pathological α-syn, primarily in the skin and/or CSF, to support the diagnosis in cases with a clinical picture suggestive of PD and an α-synucleinopathy. Certain forms of PD do not show pathological α-synuclein accumulation, such as those associated with LRRK2, RAB39B or PRKN mutations. [81] Therefore, in the presence of a clinical picture typical of PD but without evidence of pathological α-syn—primarily as assessed by SAA and iIF—a genetic disorder should be suspected. Furthermore, in the diagnostic workup, the role of assays for pathological α-syn should precede the use of nigrostriatal DaTSCAN, given the higher cost and lower specificity of the latter. We propose reserving DaTSCAN for patients who test negative for pathological α-syn and who do not show a good response to levodopa.

We suggest to incorporate into the diagnostic criteria for the various forms of α-synucleinopathy iIF and SAA. Currently, the diagnostic criteria for these diseases are based exclusively on clinical signs and symptoms, as well as surrogate tests, but do not include examinations that directly reflect the specific underlying pathology.

It is nevertheless important to emphasize that, for a broad implementation of these assays in clinical practice, specialized laboratories are required, as well as the necessary regulatory approval and overcoming the technical issues highlighted in the specific sections.

### Prognostic use

The use of specific biomarkers is particularly important for monitoring the progression of an underlying disease helping to track the disease burden. From this perspective, the various assays used to detect pathological α-synuclein have not yet been consistently applied for this purpose. Nonetheless, this aspect has recently been explored in a number of scientific papers. iIF demonstrated high diagnostic accuracy for PD even years before patients met the clinical diagnostic criteria or had received an incorrect diagnosis, in a blinded, multicenter, prospective follow-up study. [82] Skin α-syn accumulations detected by iIF were also associated with a higher incidence of RBD, as well as with greater motor and non-motor involvement, supporting the association between cutaneous deposits and a worse prognosis. [82]

Kinetic measures of α-syn-SAA were recently analysed as prognostic measures in PD. Faster seeding kinetics, particularly time to threshold (TTT), are found in GBA1-Parkinson’s disease and predict cognitive decline in PD. [83, 84] Furthermore, studies in prodromal cases showed that those individuals having faster SAA kinetics progress earlier to PD. [85]

### Clinical trials

The lack of use of a specific biomarker for α-synucleinopathies is a key factor contributing to errors in patient selection, which—especially when study cohorts are small—can compromise the final outcome of the research. The use of specific assays for pathological α-syn will enable the selection of more homogeneous patient groups, making study outcomes clearer and more interpretable. [86]

## Conclusion and future remarks

This review provides an in-depth comparison of technical aspects and diagnostic accuracies of the main techniques currently used to detect pathological α-syn as a biomarker for α-synucleinopathies. In particular, we have emphasized that, so far, the best diagnostic accuracy was found for the iIF-based detection of *p*-α-syn in skin biopsy and the SAA-based detection of pathogenic α-syn in CSF (Table 1).

**Table 1:** Main characteristics of the methods used to detect pathological α-synuclein

Assay	Sample type	Application	Diagnostic α-syn target	Comments on diagnostic accuracy	Invasiveness	Side effects	References
**ELISA**	*CSF*			*High sensitivity but low specificity (especially for total α-syn)*	High	Lumbar-puncture related (bleeding, infection, headache)	
		PD	total α-syn	Reduced total α-syn in PD but inconsistent results across cohorts			14
		PD	oligomers	Slightly higher α-syn levels in PD			15
		PD vs MSA, control	p-α-syn	Higher levels in PD compared to MSA and control but no difference in another study			16, 21
		DLB and MSA vs control	total α-syn	Lower α-syn levels vs control			19
		PD, PDD vs control, DLB	total α-syn	Lower α-syn levels vs control			20
	*Blood*			*Conflicting results*	Low	Sample collection-related (hematoma)	
		PD and MSA vs control	total α-syn	Both higher and lower total α-syn levels in PD vs controls			18, 23
		PD	oligomers	Higher levels in PD in one study, no difference in others			22, 24–25
		PD	p-α-syn	Higher levels in PD in two studies, no difference in another study			26–28
	*Extracellular vescicles*	PD	total α-syn	Increased α-syn levels in one study not confirmed in other studies	Low		30, 31
**PET/SPECT**				*Different high-affinity ligands showed promising results, but failure of trials conducted so far because* α-*syn is intracellular*	Low	Infusion of tracer-related	
		MSA/PD/DLB		Different binding reported in several parkinsonian syndromes			34–35-36
**IHC**	*Skin*		p-α-syn	*Simple and fast, but low sensitivity and does not allow for colocalization studies*	Low-to-moderate	Biopsy-related (bleeding, infection)	37–39
**iIF**	*Skin*			*High sensitivty and even higher specificity, qualitative analysis with colocalization studies (quantitative in development)*	Low-to-moderate	Biopsy-related (bleeding, infection)	
		PD	p-α-syn	p-α-syn identified in autonomic skin nerve fibers			4, 40–43, 49, 50
		MSA	p-α-syn	p-α-syn identified in both nerve fibers and Remak cells			7
		Prodromal stages (e.g., iRBD)	p-α-syn	p-syn detected in iRBD patients later phenoconverting			51–52
		DLB	p-α-syn	High specificity promising in differential diagnosis of cognitive decline			82
**SAA**	*CSF*			*High sensitivity and specificity and inter-laboratory agreement; qualitative analysis (quantitative in development)*	High	Lumbar-puncture related (bleeding, infection, headache)	
PMCA/RT-QuIC techniques		PD	seed aggregates	SAA discriminates between PD and controls; speed of α-syn aggregation may correlate with rate of phenoconversion			67–68
		LBD vs MSA	seed aggregates	Properties of α-syn aggregates differ in LBD vs MSA			69–70
		Prodromal forms (RBD, PAF, MCI)	seed aggregates	Identification of prodromal forms of synucleinopathy			63–66, 68
	*Other (skin, olfact, saliva, blood)*		seed aggregates	Promising but not validated yet	Low-to-moderate	Biopsy or procedure-related	72–76

**PDD** = Parkinson’s disease with dementia; **IHC** = immunohistochemistry; **iIF** = indirect immunofluorescence; ***p*-α-syn** = phosphorylated α-synuclein at serine 129; **SAA** = Seed Amplification Assay; **PMCA** = Protein Misfolding Cyclic Amplification; **RT-QuIC** = real time-quaking induced conversion

Importantly, iIF and α-syn-SAA have the ability to highlight different aspects of the abnormal aggregates of α-syn since iIF may detect the morphology of intraneural or intraglial aggregates in skin samples, whereas α-syn-SAA can determine their seeding activity. Thus, accumulating more comparative data to establish whether the two methods provide different or complementary information would be important. It remains to be investigated which methodology may provide the best diagnostic performance in particular pathological situations or in cases with co-pathology (Table 2). Their respective value on evaluating the clinical evolution of the underlying disease or the effect of targeted disease-modifying therapies may also be different and/or complementary. Only a few studies have investigated the correlation between skin iIF and α-syn-SAA. In the first publication, both iIF and α-syn-SAA demonstrated a high diagnostic accuracy in disclosing abnormal α-syn, although iIF displayed the better value in all variants of α-synucleinopathies. Importantly, these techniques presented a good level of agreement in identifying disease. [47] Subsequently, it was found that α-syn-SAA showed a higher sensitivity but a lower specificity than iIF for the detection of misfolded α-syn in patients with iRBD and PD. [51, 52]

**Table 2:** Important pending questions for the future

⇒Will it be possible in the future to identify pathological markers for α-synucleinopathies in easily accessible biological fluids such as saliva, tears, urine, or blood for clinical diagnostic purposes?
⇒Will the techniques used to identify misfolded synuclein in vivo, particularly indirect immunofluorescence and SAA, be standardized to allow the use of comparable data across different laboratories?
⇒Can skin biopsy and SAA be combined to increase certainty of diagnosis?
⇒Do diagnostic tests that detect pathological α-synuclein have the same accuracy in the early and advanced stages of a α-synucleinopathy?
⇒Can the search for a diagnostic biomarker in synucleinopathies also reflect clinical involvement and thus serve as a prognostic biomarker?
⇒Are the different biomarkers used to identify misfolded synuclein correlated with each other, or do they identify different aspects of the underlying pathological substrate?
⇒Are diagnostic tests for misfolded α-synuclein relevant for identifying co-pathology in other neurodegenerative diseases such as Alzheimer’s disease?
⇒Can the search for a specific α-synuclein biomarker also be used to clarify the underlying pathogenesis of these diseases?
⇒Can the tests for detection of misfolded α-synuclein identify distinct synucleinopathies (e.g. PD vs MSA vs DLB)?
⇒Can pathological biomarkers of α-synucleinopathies be used to assess the effectiveness of a disease-modifying therapy?

Finally, this review has emphasized the main current limitations and technical problems that prevent a large-scale standardization of methods to detect misfolded α-syn as a diagnostic biomarker. We have also discussed which aspects need to be improved or clarified in order to enhance the diagnostic usefulness of these methods (Table 2).

## Data Availability

The datasets generated and analysed during the current study are available from the corresponding authors on request.

## Abbreviations

α-syn: α-synuclein
p-α-syn: Phosphorylated α-synuclein at serine 129
CSF: Cerebrospinal fluid
PD: Parkinson’s disease
DLB: Dementia with Lewy bodies
PAF: Pure autonomic failure
PNS: Peripheral nervous system
CNS: Central nervous system
AD: Alzheimer’s disease
RBD: Rapid eye movement sleep behavior disorder
ELISAs: Enzyme linked immunosorbent assays
PET: Positron emission tomography
IMC: Immunohistochemistry
iIF: Indirect immunofluorescence
DC: Distribution coefficient
SAA: Seed Amplification Assay
PMCA: Protein Misfolding Cyclic Amplification
RT-QuIC: Real time-quaking induced conversion
